# Letter to the editor regarding “Development of a machine learning-based predictive model for long-term adverse outcomes in neonatal bacterial meningitis”

**DOI:** 10.1016/j.jped.2026.101523

**Published:** 2026-03-13

**Authors:** Rafael Gomes de Melo D’Elia, Angélica Saiuri de Aurélio Penteado

**Affiliations:** Hospital das Clínicas da Faculdade de Medicina da Universidade de São Paulo (HCFMUSP), São Paulo, SP, Brazil

*Dear Editor,*

We have read with great interest the manuscript [1] describing a machine-learning model to predict long-term adverse outcomes after neonatal bacterial meningitis. The topic is highly relevant: anticipating which infants will develop neurodevelopmental sequelae would meaningfully inform early follow-up, rehabilitation planning, and family counseling. The authors are to be commended for focusing on a clinically coherent cohort of term neonates enrolled over a clearly defined period and for following patients through a prespecified endpoint, producing an analytic sample with explicit accounting of exclusions and losses to follow-up (final *N* = 139 after exclusions and ≈21 % lost to follow-up).

From a clinical perspective, the study presents several notable strengths. The candidate predictors are largely derived from routine clinical evaluation, cerebrospinal fluid analysis, and neuroimaging, supporting potential applicability in everyday neonatal practice rather than reliance on experimental biomarkers. The authors also appropriately compare multiple modeling strategies and, importantly, report not only discrimination metrics but also calibration indices, Brier scores, and decision-curve analysis. Such comprehensive reporting aligns with contemporary recommendations that prognostic performance should be judged by overall clinical usefulness rather than area under the curve alone, as good discrimination does not guarantee reliable risk estimation at the bedside [2].

The emphasis on interpretability through SHAP analysis further strengthens the manuscript, as it allows clinicians to understand how familiar variables — such as cerebrospinal fluid white blood cell count, protein concentration, and the presence of seizures — drive risk estimates. This attention to transparency is particularly welcome given growing concerns about the “black-box” nature of many machine learning approaches in medicine.

Nevertheless, several issues deserve clarification before the model is considered for clinical use. First, the study is limited by its single-center design and modest final sample size, with a substantial proportion of initially eligible infants lost to follow-up. Internal train-test splitting, while informative, cannot substitute for external geographic or temporal validation to assess transportability across different referral patterns, microbiologic landscapes, and care environments. Established guidance consistently emphasizes that external validation is essential to avoid overly optimistic performance estimates and to understand likely heterogeneity in real-world settings [3].

Closely related is the trade-off the authors themselves note between discrimination and calibration. Logistic regression showed very high AUROC but poor calibration (Brier ≈0.235), while the random forest had similar discrimination with markedly better Brier score (≈0.123). Because prognostic models are often used to inform counseling and downstream clinical decisions, calibration is arguably as important as discrimination. Current frameworks for model evaluation stress that miscalibrated predictions may be clinically misleading even when ranking performance is strong, reinforcing the need for calibration plots, threshold-specific net benefit reporting, and, where appropriate, recalibration strategies prior to implementation.

Third, additional clarity regarding model development would enhance reproducibility and clinical trust. Transparent reporting standards such as the TRIPOD statement, and its recent extension for artificial intelligence–based prediction models, recommend explicit reporting of model parameters, coefficients, or feature weights, and sufficient detail to allow independent validation or clinical deployment. Adherence to these guidelines would substantially strengthen the manuscript and facilitate responsible reuse of the proposed model [4].

Relatedly, the approach to feature selection, missing data, and class imbalance deserves further exploration. While the authors’ consensus-based feature selection strategy may improve stability, it may also favor more complex algorithms. In addition, exclusion of variables with substantial missingness and limited discussion of alternative imbalance-handling strategies leave open questions about residual bias. Recent methodological work cautions that commonly used imbalance correction techniques can inadvertently harm calibration and clinical usefulness if not carefully evaluated, underscoring the importance of sensitivity analyses focused on overall predictive performance rather than discrimination alone [5].

Finally, the composite outcome mixes objective endpoints (death, abnormal imaging) with assessments that can be subjective or heterogeneous (developmental testing and telephone follow-up). While trained assessors are reported, inter-rater reliability statistics and the distribution of outcome components would aid interpretation and help determine whether the model predicts neurobiologic injury per se or, in part, follow-up ascertainment patterns.

In summary, this work has promising clinical relevance and uses commendable transparency in many analytic choices. Before translation into practice, I would encourage the authors to share model details per TRIPOD guidance (coefficients or an implementable calculator, calibration plots, and decision-curve thresholds), to report sensitivity analyses for missing data and class imbalance, and to pursue external validation in a multicenter cohort as the authors themselves recommend. These steps would make a valuable tool far more reliable for clinicians and families confronting the long-term consequences of neonatal meningitis.

## Data availability statement

N/A.

## Declaration of competing interest

The authors declare that the article content was composed in the absence of any commercial or financial relationships that could be construed as a potential conflict of interest.
